# Pneumorrhachis Secondary to Klebsiella pneumoniae Gas-Forming Paraspinal Abscess: A Case report and Review of Literature

**DOI:** 10.7759/cureus.45851

**Published:** 2023-09-24

**Authors:** Kosasih Sumitro, Dewi Norwani Basir, Adli Metussin, Kian Chai Lim, Vui Heng Chong

**Affiliations:** 1 Internal Medicine, Raja Isteri Pengiran Anak Saleha Hospital, Bandar Seri Begawan, BRN; 2 Department of Radiology, Raja Isteri Pengiran Anak Saleha Hospital, Bandar Seri Begawan, BRN

**Keywords:** gas-forming abscess, klebsiella pneumoniae (kp), sepsis, pneumorrhachis, paraparesis

## Abstract

Pneumorrhachis is a rare entity, where air pockets are found in the spinal canal and the etiology can be categorized into traumatic and non-traumatic, the latter further categorized into spontaneous, iatrogenic, and associated with infections. Infective causes are often associated with gas-forming organisms and are associated with significant morbidity and mortality. Often the diagnosis is not suspected until imaging is done. We report the case of a 57-year-old man who presented with fever, backache, lower leg weakness, and dysuria. A computed tomography scan for evaluation of intra-abdominal sepsis incidentally showed pneumorrhachis affecting the thoracic and lumbar levels, gas-forming paraspinal abscess, prostate abscess, liver cirrhosis, and sigmoid colon carcinoma. Blood culture isolated *Klebsiella pneumoniae*. The patient recovered after six weeks of intravenous antibiotics followed later by sigmoid colectomy and chemotherapy. A literature review identified 63 cases of pneumorrhachis associated with infections and can be categorized into infections with spontaneous pneumorrhachis (predominantly respiratory tract infections), infections with pneumorrhachis (predominantly with emphysematous infections), and iatrogenic with infections and pneumorrhachis (predominantly postspinal interventions). Infections with pneumorrhachis occurred in older age groups and were associated with higher mortality compared to the other two categories.

## Introduction

Pneumorrhachis where free air is present in the spinal canal (intradural or extradural) considered a rare condition and the etiologies can be categorized into traumatic and non-traumatic, the latter further categorized into spontaneous, iatrogenic, and infections related [1]. Prognosis depends on the underlying etiology and diagnosis is often only made after imaging of the spine. Infective causes are often associated with gas-forming infections affecting structures adjacent to the spine and require aggressive treatment given the high mortality rate [2]. We report the case of a 57-year-old man who presented with fever, backache, lower leg weakness, and dysuria and was diagnosed with pneumorrhachis associated with *Klebsiella pneumoniae* gas-forming paraspinal abscess. A literature review of pneumorrhachis associated with infections is presented.

## Case presentation

A 57-year-old man was admitted to the medical unit with fever, back pain, progressive bilateral leg weakness, and dysuria. He denied any gastrointestinal symptoms, trauma, or recent medical interventions. At presentation, he was febrile (38.5°C), tachycardic (110 beats per minute), and hypotensive 95/60 mmHg. He also had bilateral lower limb weakness (power 3/5) without any sensory disturbances. The rest of the neurological examinations including the upper limbs were normal. Chest, precordial, and abdominal examinations were also unremarkable. Per rectal examination revealed a tender enlarged prostate but no mass or stool abnormality.

Blood investigations during admission are shown in Table 1. The chest radiograph was normal.

**Table 1:** Baseline blood investigation during admission.

	Result	Normal Range
White cell count	18.2 x 10^3^ µL	4.2 - 12.6 x 10^3^ µL
Hemoglobin	12.5 g/dL	13.5 - 17.9 g/dL
Platelet	56,000 x 10^9^	174 - 430 x 10^9^
C-reactive protein	22.6 mg/dL	< 0.49 mg/dL
Bilirubin	121 µmol/L	3.4 - 20.5 µmol/L
Albumin	21 g/L	35-51 g/L
Creatinine	184 µmol/L	63.6 - 110.5 µmol/L
Urea	20.3 mmol/L	3.0 - 9.2 mmol/L

**Figure 1:**
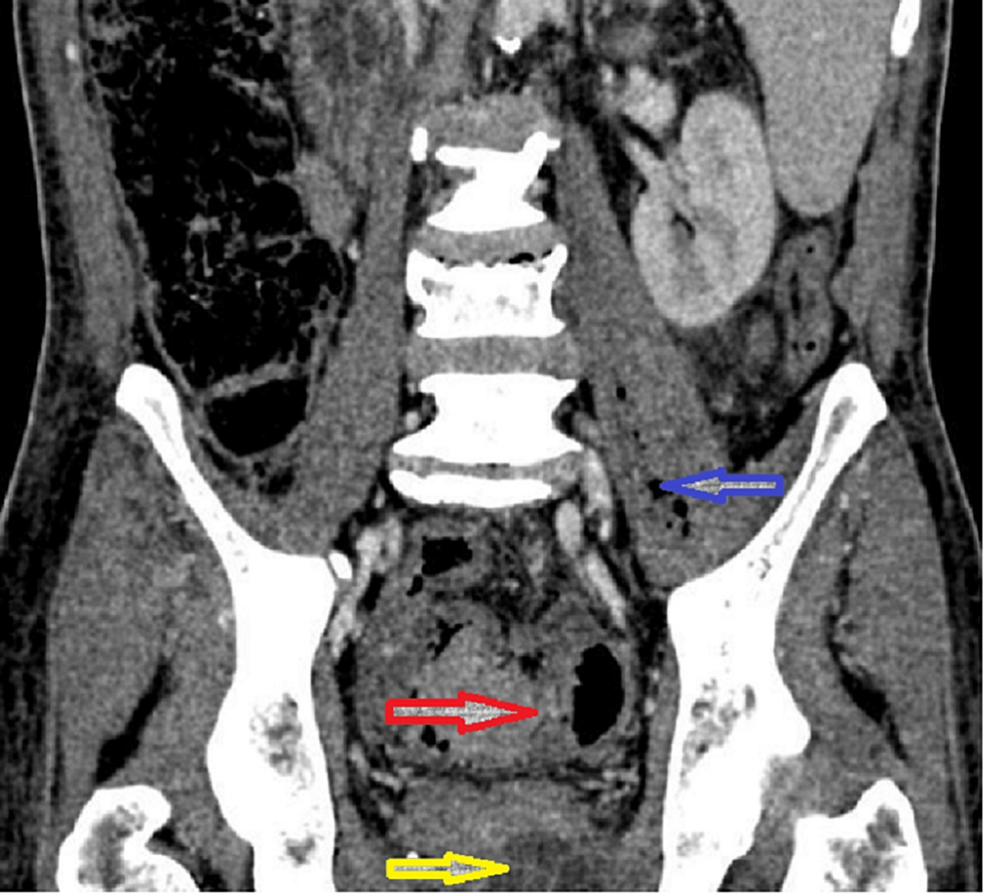
Coronal contrast-enhanced CT image Anterior coronal image of the same CT scan shows a sigmoid mass (red arrow), part of the prostate abscess (yellow arrow), and a left iliopsoas muscle abscess containing air (blue arrow).

**Figure 2:**
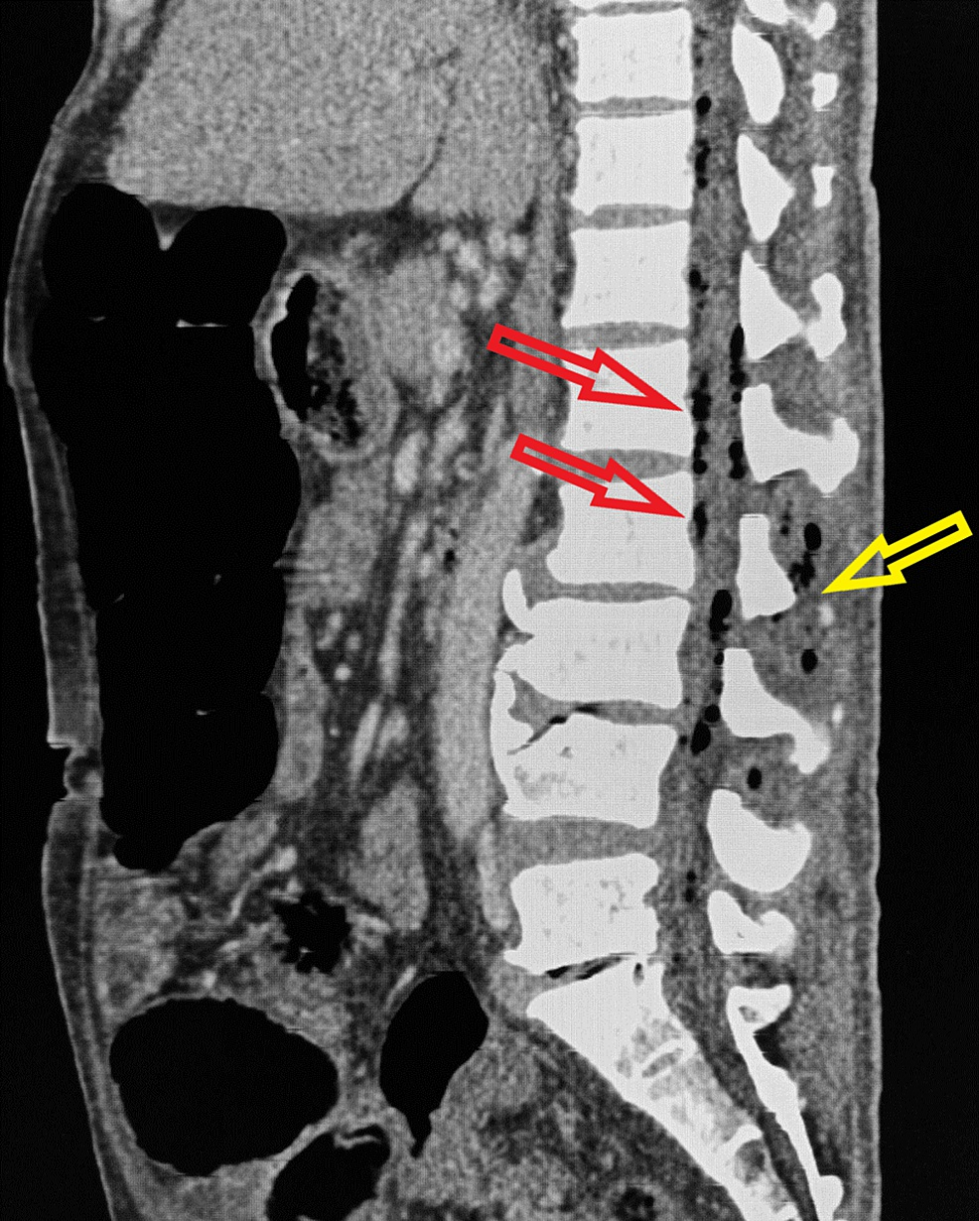
Sagittal contrast-enhanced CT image Show air pockets in the intraspinal epidural space (epidural emphysema) (red arrows) and air pockets in the lower paraspinal muscle on both sides (paraspinal abscess) (yellow arrow).

The patient’s condition deteriorated and he was transferred to the intensive care unit for management of septic shock and was started on intravenous fluid resuscitation and broad-spectrum intravenous antibiotic (piperacillin-tazobactam 1 g tds). Both urine and blood cultures isolated *K. pneumoniae* that was only resistant to ampicillin and sensitive to other antibiotics including piperacillin-tazobactam. A computed tomography (CT) scan of the abdomen and pelvis showed a prostate abscess, sigmoid mass, and liver cirrhosis with ascites (Figure 1). Interestingly, it also showed multiple air pockets in the spine in the epidural space (epidural emphysema) (Figure 2) resulting in a diagnosis of pneumorrhachis associated with emphysematous paraspinal *K. pneumoniae* infection.

Evaluation for liver cirrhosis was negative for hepatitis B (HBsAg), autoimmune or metabolic causes, but was positive for hepatitis C virus (HCV) IgG. HCV was positive with an RNA viral load count of 2,890,000 IU.

**Figure 3:**
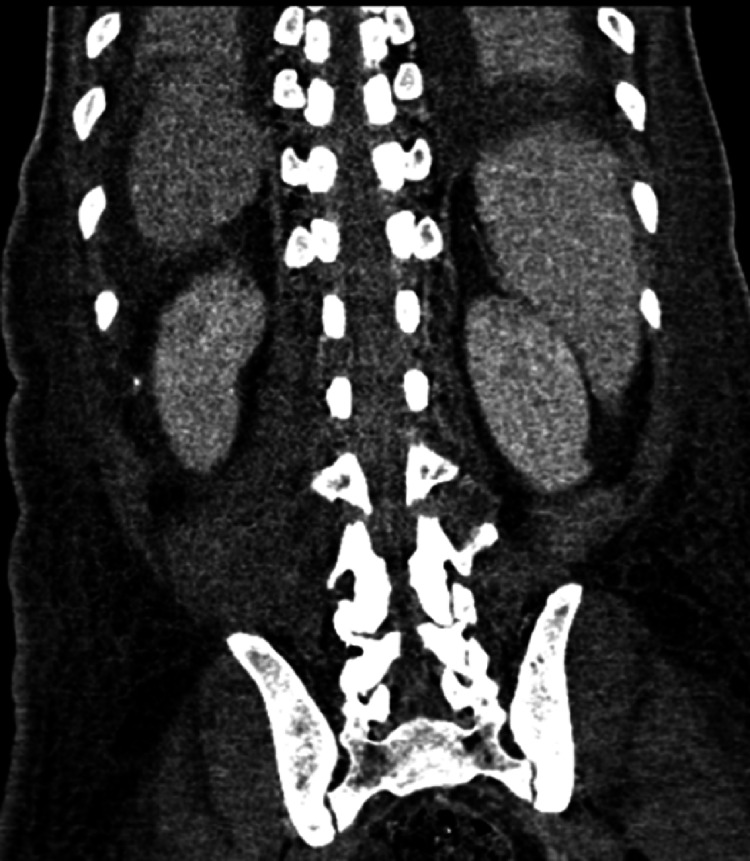
CT image follow-up Follow-up coronal CT image three weeks after broad-spectrum intravenous antibiotics showing interval resolution of the intraspinal abscess and paraspinal abscess with resolution of the pneumorrhachis.

His condition improved and he was later transferred to the medical ward. A follow-up CT scan done after three weeks of intravenous antibiotics showed resolution of pneumorrhachis and paraspinal abscesses (Figure 3) and reduction in the size of the prostate abscess. A colonoscopy showed a circumferential sigmoid tumor and histology confirmed sigmoid carcinoma (moderately differentiated adenocarcinoma). Gastroscopy for variceal screening confirmed the presence of esophageal varices and portal hypertensive gastropathy. He recovered well after six weeks of intravenous antibiotics, without any neurological deficit.

He was later referred for further treatment of his sigmoid carcinoma and later proceeded with sigmoid colectomy followed by chemotherapy. He was also treated for his chronic hepatitis C with a three months course of sofosbuvir and ribavirin. A posttreatment check showed undetectable RNA (<15 IU/mL) indicating sustained viral response and a cure.

## Discussion

Pneumorrhachis is rare and occurrences vary with etiologies. To date, it has only been reported as case reports or case series, with most reported being spontaneous or in association with trauma. The exact incidence of the pneumorrhachis is unknown. One study looking at 242 patients with pneumomediastinum reported presence of pneumorrhachis in 5.8%, and risk correlated with severity of pneumomediastinum [3]. In a study looking at trauma cases treated in a university hospital in Germany over a 13-year period (2004 and 2016), only eight cases were identified from a radiology database [4]. To date, there is no data on the incidence of pneumorrhachis-associated infections. A PubMed literature search identified 63 cases and pneumorrhachis associated with infections can be divided into three categories: a) infections resulting in spontaneous pneumorrhachis due to barotrauma (N=33 cases), typically respiratory tract infections; b) infections associated with pneumorrhachis (N=27 cases), typically emphysematous infections; and c) iatrogenic associated with infections and pneumorrhachis (N=5 cases) (Table 2). These infections include respiratory (upper and lower) tract infections complicated with the development of pneumomediastinum and surgical emphysema, emphysematous infections (i.e., osteomyelitis, pyelonephritis, and cystitis), infected sacral sores, and infections post-procedures, typically spine related. The number of reports has increased in all categories, due to an increase in occurrence and awareness with 50% of the cases of infections associated with pneumorrhachis, reported in the last five years [5-18].

**Table 2:** Sites of infection and types of organisms associated with pneumorrhachis (N=64 cases including the present case). ^a^meningitis (clinical and cerebrospinal fluid analysis consistent with meningitis but negative organism isolated) ^b^Include adenovirus (n=1), rhinovirus (n=1), respiratory syncytial virus (n=4), influenza (n=3), and human herpesvirus-6 (n=1) in an immunocompromised patient ^c^*E. coli* includes a case of meningitis

	Overall (N=64) n (%)	Infection with spontaneous pneumorrhachis (n=31) n (%)	Infection with pneumorrhachis (n=28) n (%)	Iatrogenic/ infection with pneumorrhachis (n=5) n (%)
Sites of infection				
Respiratory	30 (47.6)	30 (96.8)	0 (0)	0 (0)
Spine	19 (30.2)	0 (0)	14 (53.8)	4 (80)
Urinary tract	6 (9.5)	0 (0)	6 (23.1)	0 (0)
Sacral sore	3 (4.8)	0 (0)	3 (11.5)	0 (0)
Vascular	2 (3.2)	0 (0)	0 (0)	1 (20)
Intraabdominal	2 (3.2)	0 (0)	2 (7.7)	0 (0)
Nervous system	1 (1.6)	1 (3.2)^a^	0 (0)	0 (0)
Organisms Involved				
SARS-CoV-2	5 (7.8)	5 (16.1)	0 (0)	0 (0)
Non COVID-19 virus^b^	10 (15.6)	10 (32.2)	0 (0)	0 (0)
Klebsiella pneumoniae	5 (7.8)	0 (0)	5 (17.8)	0 (0)
Escherichia coli	9 (14.1)	0 (0)	9 (32.1)^c^	0 (0)
Clostridium species	3 (4.6)	0 (0)	2 (7.1) (*C. septicum*)	1 (20) (*C. perfringens*)
Citrobacter koseri	1 (1.5)	0 (0)	1 (3.5)	0 (0)
Morganella morganii	1 (1.5)	0 (0)	1 (3.5)	0 (0)
Others bacteria	2 (3.1)	2 (6.4)	0 (0)	0 (0)
Staphylococcus species	3 (4.6)	1 (3.2)	1 (3.5)	1 (20)
Streptococcus species	3 (4.6)	0 (0)	2 (7.1)	1 (20)
Enteric bacteria	3 (4.6)	0 (0)	3 (10.7)	0 (0)
Pneumocystis jirovecii	1 (1.5)	1 (3.2)	0 (0)	0 (0)
Fusobacterium necrophorum	1 (1.5)	0 (0)	1 (3.5)	0 (0)
Not stated	18 (28.1)	12 (38.7)	4 (14.2)	2 (40)

The pathogenesis of pneumorrhachis associated with infections depends on the categories as described. Infections with spontaneous pneumorrhachis are typically respiratory tract infections associated with barotrauma. Air leak and tracking along the vascular airway bundles eventually reach the spinal canal via the neural foramen to cause pneumorrhachis. Hence, pneumorrhachis with pneumomediastinum and surgical emphysema are common in this category [5,14]. Air can also track upward along the spinal canal into the cranium to cause pneumocephalus. In the second category, pneumorrhachis is primarily due to emphysematous infections affecting structures close to the spinal canal (vertebrae, paraspinal muscles, and retroperitoneal organs). Infections of structures away from the spine such as gastrointestinal tract (intra-abdominal sepsis) or infection complicating vascular line placement have also been reported [7,13,15,17,18]. Finally, the development of a fistula complicating infected sacral sores or malignancies (i.e., rectal tumor) can lead to CSF leak which can create a negative pressure effect resulting in air being sucked into the spinal column. Infections can also ascend the fistula and result in pneumorrhachis.

The organisms reported in the data included viruses (typically respiratory viruses) with the most common being SARS-CoV-2 which causes COVID-19, followed by respiratory syncytial virus and influenza virus, and bacteria. The most common bacteria is *E. coli* which is known to be associated with emphysematous infections, followed by Klebsiella species, Clostridium species, Staphylococcus species, Streptococcus species, Citrobacter species, Morganella species, and enteric organisms. In our case, the infection was caused by *K. pneumoniae* which is also known to cause emphysematous infections such as gas-forming pyogenic liver abscess [19]. The breakdowns of sites of infections and also underlying organisms are shown in Table 1. It is important to note that the organisms reported with these infections are some of the commonly encountered organisms in our daily clinical practices.

Interestingly, there are differences between the categories as shown in Table 3. Patients with infections and spontaneous pneumorrhachis were significantly younger (18.7 +/- 16.7 years old) compared to the other two categories (59.3 +/- 19.9 years old and 57.6 +/- 14.2 years old). This is consistent with the type of infections which were mainly due to respiratory tract viral infections. The other two groups were all bacterial infections. Overall, there were more males affected compared to females, also seen in the infections with spontaneous pneumorrhachis category but almost equal in the infection with pneumorrhachis category and slightly more females in the last category, although very few cases. All levels of the spinal column can be affected. The thoracic level was most commonly affected (71.4%) followed by the lumbar (50.8%) and the cervical (33.3%) levels with overlapping levels of involvement being common. Cervical and thoracic involvements were common in infections with spontaneous pneumorrhachis category, while in infections with pneumorrhachis category, the lumbar and thoracic levels were more commonly affected. Similarly, associated manifestations also varied with categories. Pneumomediastinum and surgical emphysema were predominantly seen in infections with spontaneous pneumorrhachis. Pneumothorax was only seen in this category. Surgical emphysema encountered in this category were mainly upper body, primarily chest and neck, and were typically moderate to large in extent whereas in the other categories, the surgical emphysema was typically localized to small areas. Pneumorrhachis associated with emphysematous infections also had structures affected; vertebrae, and surrounding paraspinal muscles [5-18].

**Table 3:** Characteristics and comparisons of demography, manifestations, and outcomes of cases of pneumorrhachis associated with infections (N=64 cases including the present case). *two cases not stated

	Overall (n=64) N (%)	Infection with spontaneous pneumorrhachis (n=31)	Infection with pneumorrhachis (n=28)	Iatrogenic/infection with pneumorrhachis (n=5)	p values
Mean age (years)	39.4 +/- 26.90	18.7 +/- 16.7	59.1 +/- 19.5	57.6 +/- 14.2	<0.001 for trend
Gender					
Male	40 (62.5)	24 (77.4)	14 (50.0)	2 (40)	0.153 for trend
Female	23 (35.9)	7 (22.6)	13 (46.4)	3 (60)	
Not stated	1 (1.6)	0 (0)	1 (3.6)	0 (0)	
Mean number of spine levels involvements	1.8 ±0.8	1.4±0.6	2.1±0.8	2.0±1.2	0.005
Level of spinal involvement*					
Cervical	21 (33.3)	12 (57.2)	6 (28.5)	3 (14.3)	0.467
Thoracic	45 (71.4)	28 (62.3)	15 (33.3)	2 (4.4)	0.013
Lumbar	32 (50.8)	3 (9.4)	25 (78.1)	4 (12.5)	<0.001
Sacrum	10 (15.9)	0 (0)	9 (90)	1 (10)	0.015
Other association					
Pneumomediastinum	35 (55.6)	31 (100)	4 (14.8)	0 (0)	<0.001
Pneumothorax	7 (11.1)	7 (22.6)	0 (0)	0 (0)	0.017
Pneumocephalus	9 (14.3)	30 (96.8)	4 (14.8)	4 (80)	<0.001
Subcutaneous emphysema	38 (60.3)	30 (96.8)	7 (25.9)	1 (20)	<0.001
Status					
Alive	43 (68.3)	26 (83.8)	13 (48.2)	4 (80)	0.039
Died	17 (62.5)	3 (9.7)	12 (44.4)	1 (20)	
Not reported	4 (6.3)	2 (6.5)	2 (7.4)	0 (0)	

Signs and symptoms of pneumorrhachis are also dependent on the underlying etiology or categories. Signs and symptoms consist of sepsis related (i.e., fever, lethargy, reduced intake, altered consciousness or delirium), symptoms specific to organs or structures involved such as respiratory symptoms (i.e., cough, shortness of breath, chest discomfort or pain), urinary symptoms (i.e., renal angle or suprapubic pain or discomfort, dysuria and polyuria), and symptoms related to pneumorrhachis (i.e., non-specific back pain or discomfort depending on level of spine affected and neurology such as paresis), and associated manifestations such as surgical emphysema and pneumomediastinum (i.e., cutaneous swelling and chest pain or discomfort, dyspnea, and tachycardia). However, symptoms of pneumorrhachis are minimal or nonspecific and pneumorrhachis are often incidentally detected after imaging. Manifestations are also dependent on the extent of spinal column involvement which correlates with the amount of air present. A review on trauma-related pneumorrhachis reported only 9% presented with symptoms directly attributed to the pneumorrhachis: sensory radiculopathy (2%), motor radiculopathy (1%), and myelopathy (6%) [20].

The mainstay of treatment is to treat the underlying infections, often with resolution of the pneumorrhachis [20]. Generally, emphysematous or gas-forming infections are often associated with higher mortality rates, 44.4% compared to 20% in the iatrogenic and infection category, and the least (9.7%) in the infections with spontaneous pneumorrhachis (Table 2). Therefore, infections need to be aggressively treated. Surgery or drainage may be required in some cases especially if there are large abscess collections and fistula. The usage of high oxygen concentration may help with the resolution of pneumorachis. Otherwise, aspiration of air pockets in the spinal column may be required if there are tension effects causing downstreaming neurology. However, in most instances, the pneumorrhachis including associated manifestations will resolve with treatment of the primary pathology.

## Conclusions

We reported an interesting case of extensive pneumorrhachis secondary to *K. pneumoniae* paraspinal emphysematous infection. Pneumorrhachis was only diagnosed after imaging of the abdomen to assess for the source of sepsis. The infection was treated with resolution of the pneumorrhachis. A review of the literature showed that pneumorrhachis associated with infections can be divided into three categories: the most common being infections with spontaneous pneumorrhachis which are typically associated with respiratory tract infections, followed by infections with pneumorrhachis which are often associated with emphysematous infections, and lastly iatrogenic with infections and development of pneumorrhachis. There are distinct differences between the three categories: younger age, concomitant pneumomediastinum and surgical emphysema, and lower mortality in the most common group, whereas older age in the other two groups with higher mortality rates.
